# Electroacupuncture pretreatment preserves telomerase reverse transcriptase function and alleviates postoperative cognitive dysfunction by suppressing oxidative stress and neuroinflammation in aged mice

**DOI:** 10.1111/cns.14373

**Published:** 2023-07-27

**Authors:** Wei Wang, Chen Chen, Qiang Wang, Ji‐Guang Ma, Yan‐Song Li, Zheng Guan, Rui Wang, Xin Chen

**Affiliations:** ^1^ Department of Anesthesiology The First Affiliated Hospital of Xi'an Jiaotong University Xi'an Jiaotong University Xi'an Shaanxi China; ^2^ Department of Anesthesiology The First People's Hospital of Foshan Foshan Guangdong China; ^3^ Department of Burns and Plastic surgery Hainan Hospital of PLA General Hospital Sanya Hainan China

**Keywords:** autophagy, Neuroinflammation, oxidative stress, postoperative cognitive dysfunction, telomerase reverse transcriptase

## Abstract

**Background:**

Elderly patients often exhibit postoperative cognitive dysfunction (POCD), a postsurgical decline in memory and executive function. Oxidative stress and neuroinflammation, both pathological characteristics of the aged brain, contribute to this decline. This study posits that electroacupuncture (EA) stimulation, an effective antioxidant and anti‐inflammatory modality, may enhance telomerase reverse transcriptase (TERT) function, the catalytic subunit of telomerase known for its protective properties against cellular senescence and oxidative damage, to alleviate POCD in aged mice.

**Methods:**

The animal POCD model was created by subjecting aged mice to abdominal surgery, followed by EA pretreatment at the Baihui acupoint (GV20). Postoperative cognitive function was gauged using the Morris water maze (MWM) test. Hippocampal TERT mRNA levels and telomerase activity were determined through qPCR and a Telomerase PCR ELISA kit, respectively. Oxidative stress was assessed through superoxide dismutase (SOD), reactive oxygen species (ROS), and malondialdehyde (MDA) levels. Iba‐1 immunostaining determined the quantity of hippocampal microglia. Additionally, western blotting assessed TERT, autophagy markers, and proinflammatory cytokines at the protein level.

**Results:**

Abdominal surgery in aged mice significantly decreased telomerase activity and TERT mRNA and protein levels, but increased oxidative stress and neuroinflammation and decreased autophagy in the hippocampus. EA‐pretreated mice demonstrated improved postoperative cognitive performance, enhanced telomerase activity, increased TERT protein expression, improved TERT mitochondrial localization, and reduced oxidative damage, autophagy dysfunction, and neuroinflammation. The neuroprotective benefits of EA pretreatment were diminished following TERT knockdown.

**Conclusions:**

Our findings underscore the significance of TERT function preservation in alleviating surgery‐induced oxidative stress and neuroinflammation in aged mice. A novel neuroprotective mechanism of EA stimulation is highlighted, whereby modulation of TERT and telomerase activity reduces oxidative damage and neuroinflammation. Consequently, maintaining TERT function via EA treatment could serve as an effective strategy for managing POCD in elderly patients.

## Graphical abstract

Preserving telomerase reverse transcriptase function can alleviate postoperative cognitive dysfunction in aged mice by reducing oxidative stress and neuroinflammation. Electroacupuncture pretreatment is an effective strategy for maintaining postoperative hippocampal telomerase reverse transcriptase function.

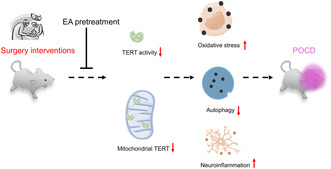

## 1 INTRODUCTION

Postoperative cognitive dysfunction (POCD), or perioperative neurocognitive disorder (PND), is a significant complication occurring postsurgery.^1^, ^2^, ^3^ Notably associated with elevated morbidity, mortality, and hospitalization rates, POCD is characterized by postsurgery reductions in learning, memory, and executive function abilities.^4^, ^5^ It poses a particularly high risk for senior patients,^4^, ^5^, ^6^ with animal studies reflecting worsened cognitive outcomes in aged subjects postsurgery.^7^, ^8^, ^9^ Thus, targeting age‐associated pathological alterations may be a viable strategy for treating POCD.

Oxidative stress, an intrinsic component of cellular aging, becomes increasingly pronounced in the brain with aging,^10^, ^11^ potentially due to a gradual decrease in traditional antioxidant defenses such as superoxide dismutase (SOD),^12^ coupled with deteriorating mitochondrial functionality.^13^ This susceptibility can result in damage to proteins, lipids, and DNA, thereby providing a possible trajectory for the onset of neurodegenerative diseases like Alzheimer's disease (AD)^14^, ^15^ and Parkinson's disease (PD).^16^, ^17^ Elevated hippocampal oxidative stress is also observed in post‐surgical aged animals.^18^, ^19^ Furthermore, oxidative stress in the aging brain is exacerbated by neuroinflammation, mediated by microglia.^20^ Surgery stress can prime hippocampal microglia activation,^7^, ^21^ with an even more pronounced effect in aged subjects.^7^ Adding another layer of complexity, oxidative imbalances tied to aging can induce the accumulation of harmful proteins and dysfunctional mitochondria.^22^ Under normal circumstances, these defective proteins and organelles would be removed by a lysosomal degradation process known as “autophagy”.^22^ However, surgical interventions can disrupt this delicate balance, interfering with hippocampal autophagy, and instigating oxidative damage.^23^, ^24^ Despite these insights underscoring the pivotal role of oxidative stress in POCD, and its intricate interconnections with neuroinflammation and autophagy, the precise mechanism underpinning surgery‐induced oxidative stress remains elusive.

Emerging evidence suggests that the maintenance of telomerase activity could be an effective defense against age‐related diseases.^25^, ^26^, ^27^ Telomerase is the exclusive enzyme that maintains and repairs telomeres (the termini of linear chromosomes) and it is also implicated in the mitigation of the aging process.^27^, ^28^ The telomerase complex includes the catalytic protein component (telomerase reverse transcriptase, TERT) and the RNA component TERC.^25^, ^26^ In the adult and aged brain, the primary activity of telomerase is confined to proliferative cells like neural stem cells (NSCs).^29^ However, TERT, the catalytic functional subunit of telomerase, is also present in postmitotic neurons where it carries out crucial noncanonical functions.^26^, ^30^, ^31^ Among these is providing an essential antioxidant mechanism, accomplished by the translocation of TERT from the nucleus to the mitochondria,^31^, ^32^ where it can reduce ROS levels and protect cells from oxidative damage.^33^, ^34^ This unique attribute of TERT suggests that preserving its function, or the activity of telomerase as a whole, could potentially lead to mitigation of POCD by curbing oxidative stress.

Building upon this understanding, innovative advancements, such as the commercially available telomerase activator (TA‐65), aim to combat the effects of aging and protect against oxidative damage.^35^, ^36^ Parallelly, electroacupuncture (EA), a technique that merges traditional acupuncture with brain stimulation methodologies,^37^ demonstrates a significant neuroprotective impact against cognitive dysfunctions related to aging. Evidence indicates that EA can alleviate cognitive dysfunction and pathological changes in a D‐galactose‐induced aging model.^38^ Clinical research further suggests that acupuncture enhances cognitive outcomes and forestalls the emergence of characteristic pathology in patients with AD and PD.^39^, ^40^ Supporting animal research echoes these findings, confirming that EA treatment alleviates cognitive deficits in mouse models of Alzheimer's and Parkinson's diseases by reducing neuroinflammation and promoting autophagy.^41^, ^42^, ^43^ Furthermore, EA‐pretreated animals exhibit diminished oxidative damage in the brain following ischemic stroke.^44^, ^45^ Despite the promising data, the specific mechanism, especially whether the antioxidant and neuroprotective effects of EA pretreatment are achieved through modulating TERT function, remains to be definitively elucidated.

Therefore, this study aims to examine the role of TERT in POCD and assess whether EA pretreatment can ameliorate POCD in aged mice by maintaining TERT function and reducing hippocampal oxidative stress.

## 2 MATERIALS AND METHODS

### 2.1 Animals

Animal study protocols and procedures were approved by Xi'an Jiaotong University, adhering to the NIH “Guide for the Care and Use of Laboratory Animals”. Male C57BL/6J mice, 20 months old and weighing between 26 and 30 g, were obtained from Xian Jiaotong University, Xi'an, Shaanxi, China. They were housed in a controlled environment with consistent temperature and humidity, and a 12‐hour light/dark cycle, and were given unrestricted access to a standard diet and water.

### 2.2 Surgical model

The mice underwent abdominal surgery under anesthesia, induced with Avertin (400 mg/kg, i.p.; 2.5% 1:1 w/v 2,2,2‐Tribromoethanol Acros Geel, Sigma). The surgical method was based on an earlier study by Rosczyk et al.,^46^ with minor modifications. Postanesthesia, mice were placed supine on a heating pad, with the abdominal area shaved and sterilized before making a 2‐cm midline incision. Sterile gauze was used to manipulate the small intestine, liver, colon, and stomach sequentially over 25 min. After this, the peritoneum, muscle wall, and skin were sequentially closed with 6–0 absorbable sutures. Sham operations involved anesthesia with Avertin but without surgery. Postoperative incision pain was minimized by injecting 1% ropivacaine (100 μL) into the incision area. To prevent postoperative infections, penicillin (80 U/g) was administered intraperitoneally immediately after the surgery, and again at 24 and 48 h postsurgery.

### 2.3 Electroacupuncture (EA) pretreatment

Mice were anesthetized using 2% isoflurane (Macklin) delivered via a nose‐cone system, followed by a steel needle insertion into the GV20 acupoint (Baihui) to a depth of 2 mm. The needle was then connected to an electrode from an electrical stimulator (G6805‐2, Qingdao Xinsheng Ltd.). The acupoint was electrically stimulated at an intensity of 1 mA and a frequency of 2/15 Hz (disperse‐dense waves) for 10 min. This EA stimulation was conducted daily, starting 3 days before surgery. Sham stimulation involved needle insertion under isoflurane anesthesia without EA stimulation.

### 2.4 Intracerebroventricular injection (i.c.v.) of TERT siRNA

After the last episode of EA stimulation, mice were maintained in 2% isoflurane anesthesia and transferred to a stereotactic apparatus (Kopf Instruments), as reported previously.^47^ A Hamilton syringe was positioned into the right lateral ventricle (stereotaxic coordinates: anteroposterior −0.5 mm, lateral from bregma +1 mm, and ventral from dura −1.6 mm). TERT siRNA (siTERT, 400 pmol, diluted in 5 μL saline, Thermo Fisher Scientific) or scrambled siRNA (negative control, diluted in 5 μL saline, Thermo Fisher Scientific) was injected through the syringe at a rate of 2 μL/min. The syringe was kept in place for an additional 5 min to allow complete siRNA diffusion.

### 2.5 Experimental groups

The experimental design of our study was divided into two parts.

**FIGURE 1:**
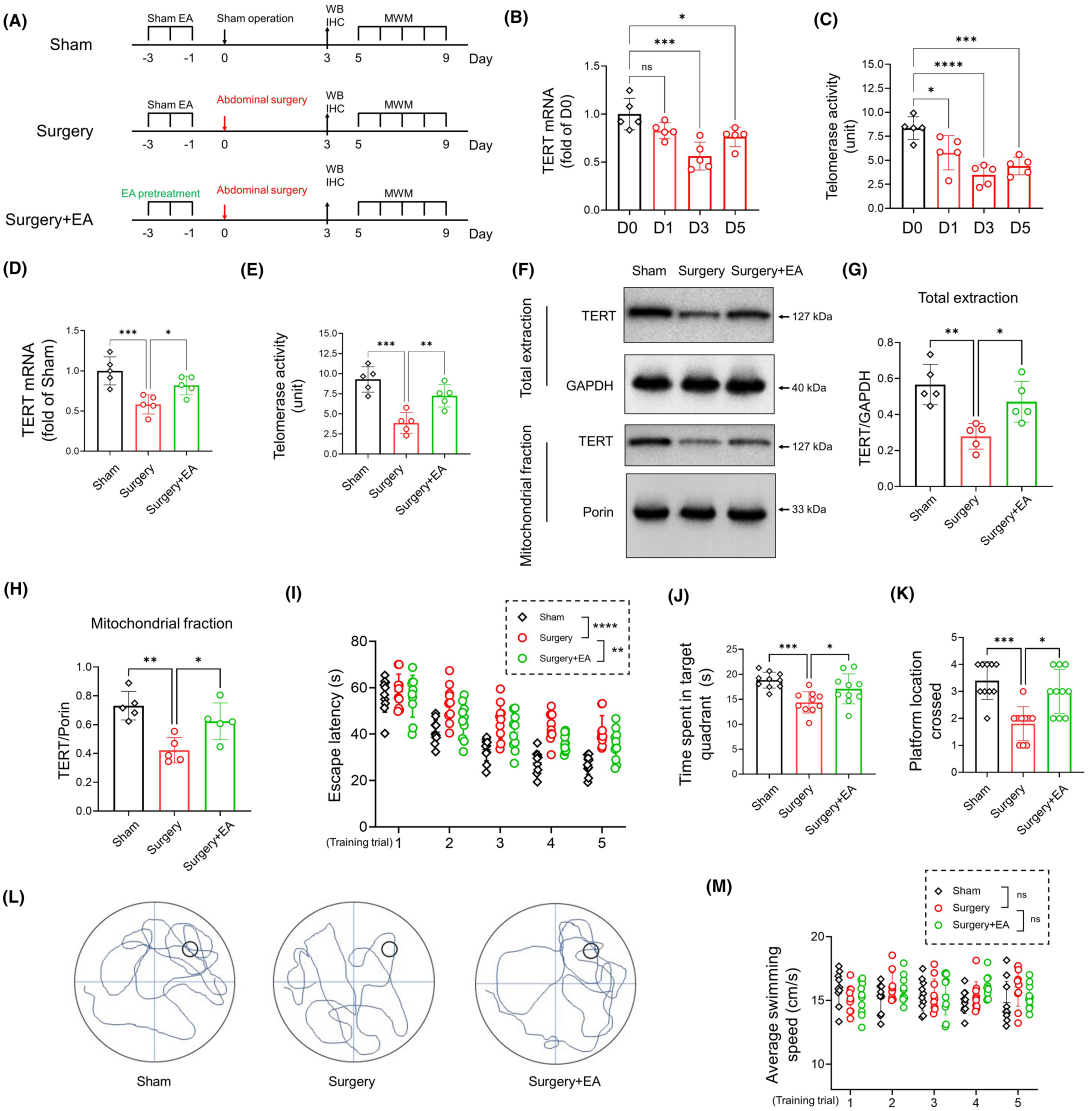
EA Pretreatment Alleviates Postoperative Cognitive Dysfunction and Enhances TERT Function in the Hippocampus of Aged Mice. (A) Schematic representation of the experimental design. (B) TERT mRNA levels determined by RT‐PCR on D0 (immediately before surgery), D1, D3, and D5 postoperative days (*n =* 5/group). (C) Telomerase activity in hippocampal tissues assessed using the TeloTAGGG Telomerase PCR ELISA kit on D0, D1, D3, and D5 (*n =* 5/group). (D) TERT mRNA analyzed on the third postoperative day (D3) for sham, surgery, and surgery+EA groups (*n =* 5/group). (E) Telomerase activity in hippocampal tissues measured on D3 for sham, surgery, and surgery+EA groups (*n =* 5/group). (F) Western blot determining TERT protein levels in total proteins and mitochondrial fractions on D3. TERT levels in total proteins and mitochondrial fractions were normalized to GAPDH and Porin, respectively. (G, H) Analysis of hippocampal TERT in total proteins and mitochondrial fractions (*n =* 5/group). (I) Escape latency during 5‐day MWM training trial for all three groups of aged mice. The surgery group exhibited longer latency to reach the hidden platform compared to sham mice, whereas EA‐pretreated mice displayed shorter escape latency (two‐way ANOVA, *n =* 10/group). (J, K) Probe trial (platform removed) performed 30 minutes after the last training trial (*n =* 10/group). The surgery group spent less time in the target quadrant and had fewer platform crossings than sham mice, whereas EA‐pretreated mice spent more time in the target quadrant and had increased platform crossings (*n =* 10/group). (L) Representative trace diagrams from the probe trial. (M) Average swimming speed of aged mice in MWM training trials (cm/s, *n =* 10/group). Data are presented as mean ± SD. *p < 0.05, **p < 0.01, ***p < 0.001, ****p < 0.0001. IHC, immunohistochemistry; MWM, Morris Water Maze; TERT, telomerase reverse transcriptase; WB, Western blot.

For Experimental Part I, mice were randomly distributed among three groups: (1) Sham group: mice underwent sham stimulation and sham surgery; (2) Surgery group: mice were subjected to sham stimulation followed by abdominal surgery; (3) Surgery+EA group: mice received EA pretreatment prior to abdominal surgery. The experimental design is detailed in Figure 1A.

Experimental Part II was composed of four groups, as outlined in Figure 4A: (1) Sham group: mice underwent sham stimulation and sham surgery, and received an intracerebroventricular (i.c.v.) injection of scrambled small interfering RNA (siRNA) 24 h before the planned surgical procedure; (2) Surgery group: mice received sham stimulation, underwent abdominal surgery, and received an i.c.v. injection of scrambled siRNA 24 h prior to surgery; (3) Surgery+EA group: mice were pretreated with EA, underwent abdominal surgery, and received an i.c.v. injection of scrambled siRNA 24 h before surgery; (4) Surgery+EA + siTERT group: mice were pretreated with EA, underwent abdominal surgery, and received an i.c.v. injection of TERT‐specific siRNA (400 pmol) 24 h before the planned surgical procedure.

### 2.6 Behavioral tests

Cognitive function postsurgery was evaluated using the Morris water maze (MWM) test, in accordance with our previous research,^48^ with slight adjustments. The mice were acclimated to the test room for 1 h prior to the test, and were given 20‐minute rest periods between trials to mitigate potential stress or fatigue. The water temperature in the swimming pool was maintained within the optimal range of 24–26°C. The MWM test training task, conducted over five consecutive days from postoperative day 5 (D5) to D9, entailed four trials per day (70‐second cut‐off; 20‐minute interval) with 10 mice per group. Both the swimming path and the escape latency, or the time it took to reach the platform, were recorded using EthoVision software (Noldus Information Technology). Four hours following the conclusion of the fifth training task, a 40‐second probe trial was performed with the platform removed. Both the time spent in the target quadrant and the number of platform crossings were noted for further analysis.

### 2.7 Detection of telomerase activity

We analyzed telomerase activity within the hippocampus using the TeloTAGGG Telomerase PCR ELISA kit (Roche Diagnostics) as instructed by the manufacturer. Initially, the collected hippocampal tissues were fragmented in liquid nitrogen and subsequently incubated with the kit's ice‐cold lysis buffer. After lysis, we harvested the supernatant for protein concentration quantification, performed using a BCA protein assay kit (Boster). Samples prepared with the reaction mixture were then subjected to a telomeric repeat amplification reaction in a thermal cycler (Applied Biosystems 7300 Sequence Detection System). During this process, telomeric repeats (TTAGGG) are appended to the 3′ ends of the biotin‐labeled primer by telomerase and then amplified through PCR. Following this, the PCR product was denatured and allowed to hybridize with digoxigenin‐labeled, telomeric repeat‐specific probes. The product was then immobilized to a streptavidin‐coated microplate via the biotin‐labeled primer. Finally, the immobilized product was detected using peroxidase‐conjugated anti‐digoxigenin antibodies. The absorbance at 450 nm, resulting from the peroxidase reaction with TMB substrates, was measured to quantify the telomerase activity.

### 2.8 Western blot and oxidative stress examination

Mice were euthanized through phenobarbital overdose (200 mg/kg, intraperitoneal), and the hippocampal tissues were promptly extracted and homogenized in ice‐cold RIPA buffer (Beyotime). Protein concentration was evaluated using a BCA protein assay kit (Boster). Based on these concentration measurements, supernatants were diluted to a 10 mg/mL protein concentration with sterile PBS (pH 7.4) and apportioned for western blot and oxidative stress evaluations, respectively (*n =* 5/group).

Mitochondrial proteins were extracted using the Tissue Mitochondria Isolation Kit (Beyotime), adhering to the manufacturer's guidelines. Briefly, we minced fresh hippocampal tissue on ice and mixed it with precooled mitochondrial separation reagent A, maintaining a ratio of 50 mg tissue to 10 times the volume of the reagent. The mixture was homogenized on ice and centrifuged at 600*g* at 4°C for 5 min to obtain the supernatant. This supernatant underwent secondary centrifugation at 11,000*g* and 4°C for 10 min to harvest the mitochondrial fraction. Western blot analysis, targeting characteristic proteins of mitochondria (cytochrome c oxidase subunit 3, COX3), nuclear (LaminB), and cytoplasmic (GAPDH) contents, was performed to validate the purity of the mitochondrial fraction (Figure S1).

Equal protein samples (40 μg) were separated by 10% sodium dodecyl sulfate‐polyacrylamide (SDS) gel electrophoresis and transferred onto polyvinylidene difluoride (PVDF) membranes (Millipore). Membranes were blocked using 5% nonfat milk in TBST or 3% BSA [for phosphorylated mammalian target of rapamycin (mTOR) at Ser2448, p‐mTOR] for an hour at room temperature, followed by an overnight incubation at 4°C with the respective primary antibodies. The following primary antibodies were used: anti‐Beclin‐1 (Cell Signaling Technology, 1: 1000), anti‐GAPDH (Proteintech, 1: 5000), anti‐interleukin (IL)‐6 (Abclonal, 1: 1000), anti‐ COX3 (Abclonal, 1: 500), anti‐LaminB (Abclonal, 1: 500), and anti‐microtubule‐associated proteins 1A/1B light chain 3B (LC3B) (Cell Signaling Technology, 1: 500), anti‐mTOR (Abclonal, 1: 1000), anti‐Porin (Abclonal, 1: 1000), anti‐p‐mTOR (Abcam, 1: 1000), anti‐TERT (Novus Biologicals, 1: 1000), and anti‐tumor necrosis factor (TNF)‐α (Abclonal, 1: 500). Then, the membranes were washed three times with TBST and incubated with HRP‐conjugated secondary antibodies (anti‐mouse or rabbit IgG, 1:5000; Boster) for 2 h at room temperature. Eventually, the protein bands were visualized utilizing enhanced chemiluminescent reagents (Beyotime) via the Fluochem HD2 Imaging System (Alpha Innotech) or captured on X‐ray film. The blot density was quantified with ImageJ software (version 1.43). The protein level of p‐mTOR was normalized to total mTOR. The mitochondrial TERT protein was normalized to Porin. LC3B‐II was normalized to LC3B‐I as in our earlier study.^49^ The values were averaged from three duplicate experiments.

We assessed superoxide dismutase (SOD), reactive oxygen species (ROS), and malondialdehyde (MDA) levels to gauge the degree of oxidative damage in the hippocampus,^19^ using commercial spectrophotometric assay kits as per the manufacturer's protocols (SOD kit, Nanjing Jiancheng Bioengineering Institute; ROS kit, DHE method, Biorab Technology; MDA kit, Beyotime). The levels of SOD, ROS, and MDA were reported as milli‐unit per milligram protein (mU/mg protein), relative fluorescence units (RFU)/mg protein, and pmol/mg protein, respectively.

### 2.9 Immunofluorescence analysis

For immunofluorescence examinations, mice underwent transcardial perfusion with 4% iced formaldehyde (*n =* 5/group). The brain was excised, paraffin‐embedded, and sectioned into 5‐μm serial slices. Following deparaffinization, rehydration, and microwave‐assisted antigen retrieval (Citrate Buffer, pH 6.0), the slices were treated with normal goat bovine serum for an hour at room temperature. This was followed by overnight incubation at 4°C with primary antibodies targeting glial fibrillary acidic protein (GFAP, an astrocyte marker) (rabbit polyclonal, abclonal; 1:400) and ionized calcium‐binding adapter molecule 1 (Iba‐1, a microglia marker) (rabbit monoclonal, Cell Signaling Technology; 1:400). The next day, slices were washed with PBS and incubated for an hour at room temperature with Dylight 488 (1:200; goat anti‐rabbit, Abcam). The nuclei were stained with 4′,6‐diamidino‐2‐phenylindole (DAPI). Images were obtained via immunofluorescence microscopy (40× objective lens, Olympus). Quantitative analyses were carried out manually by enumerating positive cells using the Image Pro™ Plus software.

### 2.10 Quantitative real‐time polymerase chain reaction (qRT‐PCR)

The total RNA was extracted from hippocampus tissues using Trizol Reagent (Invitrogen) following the manufacturer's protocol (*n =* 4 or 5/group). For TERT mRNA detection, total RNA (2 μg) was reverse transcribed into cDNA with AMV Reverse Transcriptase (Promega). GAPDH was used as the loading control. The qRT‐PCR assay was conducted on the Applied Biosystems 7300 Sequence Detection System using the SYBR Green master mix (Applied Biosystems). The primers were purchased from RiboBio and listed as follows: TERT, Forward: 5’‐CTGCGTGTGCGTGCTCTGGAC‐3′, Reverse: 5’‐CACCTCAGCAAACAGCTTGTTCTC‐3′; GAPDH, Forward: 5’‐TGTGGGCATCAATGGATTTGG‐3′, Reverse: 5’‐ACACCATGTATTCCGGGTCAAT‐3′. Fold change of TERT mRNA was determined by a comparative threshold cycle (Ct) method^50^ with the formula 2^−ΔΔCt^.

### 2.11 Statistical analysis

The data collected were delineated as the mean ± standard deviation (SD). A normality test was conducted on all variables employing the Shapiro–Wilk methodology. In relation to the MWM examinations, the escape latency data were subjected to a two‐way Analysis of Variance (ANOVA), succeeded by a post hoc analysis with Dunn's multiple comparisons tests. The data corresponding to platform crossing times, which showed deviation from a normal/Gaussian distribution, underwent analysis through the Kruskal–Wallis ANOVA test. Comparative analysis for other groupings was carried out using a one‐way ANOVA, followed by Dunnett's multiple comparisons test. These analyses were performed utilizing GraphPad Prism 5 (GraphPad Software Inc.). A *p*‐value of less than 0.05 was deemed to denote statistical significance.

## 3 RESULTS

### 3.1 EA pretreatment mitigates postoperative cognitive dysfunction and preserves TERT function in aged mice's hippocampus

To assess surgical stress's effect on hippocampal TERT function, we measured TERT mRNA and telomerase activity in the hippocampus right before surgery (D0), 1 day (24 h) postsurgery (D1), D3, and D5. A significant decrease was observed in TERT mRNA levels and telomerase activity postsurgery (Figure 1B,C; TERT mRNA, *p <* 0.001 for D3, *p =* 0.0247 for D5; telomerase activity, *p =* 0.0157 for D1, *p <* 0.0001 for D3, *p <* 0.001 for D5; *n =* 5/group), compared to D0. The lowest levels of TERT mRNA and telomerase activity on D3 led us to select this day for subsequent laboratory tests.

The reduced TERT mRNA level and telomerase activity improved in EA‐pretreated mice (Figure 1D,E, *surgery* vs. *surgery + EA*, *p =* 0.0366 for TERT mRNA, *p <* 0.01 for telomerase activity, *n =* 5/group). Additionally, there was a significant decrease in the hippocampal TERT amount in both total and mitochondrial proteins in the surgery group compared with sham mice (Figure 1F–H, *p <* 0.01 and *p <* 0.01 for total and mitochondrial TERT, *n =* 5/group). However, EA‐pretreatment mitigated this postsurgery decrease in total and mitochondrial TERT protein (Figure 1F–H, *surgery* vs. *surgery + EA*, *p =* 0.0194 for total TERT, *p =* 0.0203 for mitochondrial TERT, *n =* 5/group).

Next, we implemented the MWM test from D5 to D9, with the experimental design depicted in Figure 1A. The surgery group exhibited increased escape latency to locate the hidden platform (Figure 1I, two‐way ANOVA, treatment condition, *F*_(1, 18)_ = 85.88, *p <* 0.0001 for treatment condition, *n =* 10/group), spent less time in the target quadrant (Figure 1J, *p <* 0.001, *n =* 9/group), and had fewer platform crossings (Figure 1K, *p <* 0.001, *n =* 10/group, Kruskal–Wallis ANOVA test) compared to the sham group, indicative of successful postoperative cognitive dysfunction induction. In contrast, EA‐pretreated mice showed improved postoperative cognitive performance, evidenced by decreased escape latency (Figure 1I, two‐way ANOVA, treatment condition, *F*_(1, 18)_ = 12.27, *p =* 0.0025 for treatment condition, *n =* 10/group), more time spent in the target quadrant (Figure 1J,L, *P =* 0.0266, *n =* 10/group), and increased platform crossings (Figure 1K,L, *p =* 0.0136, *n =* 10/group, Kruskal–Wallis ANOVA test). No statistically significant differences were found in the average swimming speed during training trials across the three experimental groups (Figure 1M, two‐way ANOVA, treatment condition, *F*_(2, 27)_ = 1.124, *p =* 0.3398 for treatment condition, *n =* 10/group).

### 3.2 EA pretreatment diminishes oxidative stress and sustains autophagy in the Hippocampus following surgery in aged mice

**FIGURE 2:**
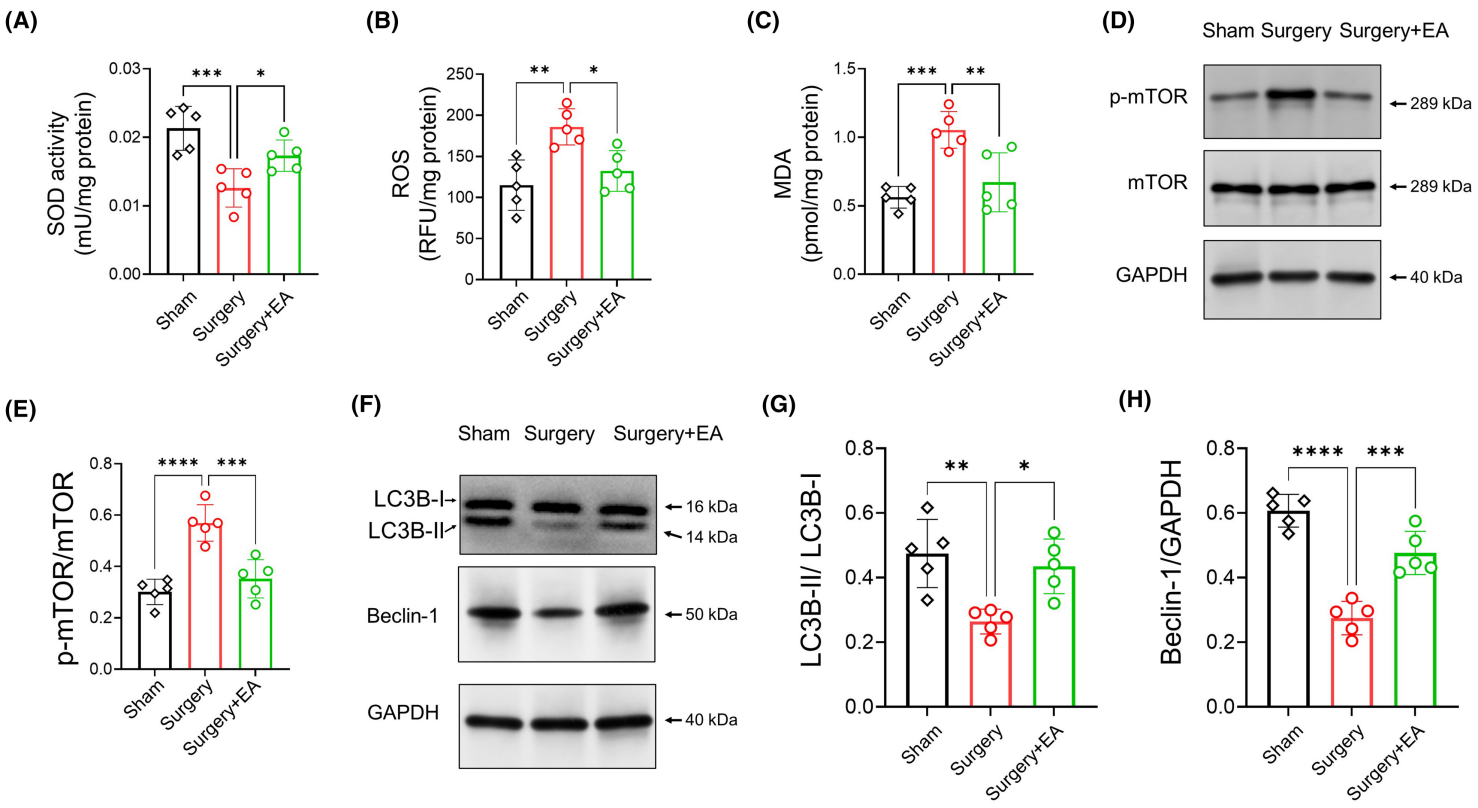
EA Pretreatment Mitigates Oxidative Stress and Autophagy Dysfunction Following Surgery in Aged Mice. (A–C) Hippocampal SOD, ROS, and MDA levels measured on D3 using commercial kits to evaluate oxidative damage in the hippocampus (*n =* 5/group). (D) Western blot analysis of phosphorylated mTOR at Ser2448 (p‐mTOR) on D3. The p‐mTOR protein level was normalized to total mTOR. The mTOR pathway is a key regulator of autophagy, with pathway activation signifying autophagy suppression. (E) Notably, p‐mTOR levels in the hippocampus significantly increased postsurgery (*sham* vs. *surgery*, *n =* 5/group), but this increase was thwarted in EA‐pretreated mice (*surgery* vs. *surgery + EA*, *n =* 5/group). (F) Western blot analysis of autophagy markers (LC3B and Beclin‐1) on D3. LC3B‐II was normalized to LC3B‐I, while Beclin‐1 protein level was normalized to GAPDH. (G, H) Surgical stress led to a decrease in both the LC3B‐II to LC3B‐I ratio and Beclin‐1 protein level in the hippocampus (*sham* vs. *surgery*, *n =* 5/group). However, EA‐pretreated mice showed recovery in the LC3B‐II to LC3B‐I ratio and Beclin‐1 protein level in the hippocampus (*surgery* vs. *surgery + EA*, *n =* 5/group). Data are represented as mean ± SD. *p < 0.05, **p < 0.01, ***p < 0.001, ****p < 0.0001. LC3B, microtubule‐associated proteins 1A/1B light chain 3B; MDA, malondialdehyde; mTOR, mammalian target of rapamycin; ROS, reactive oxygen species; SOD, superoxide dismutase.

Given the role of increased oxidative stress in POCD development,^18^ we evaluated the concentration of SOD (an antioxidant enzyme), ROS, and MDA (an oxidative stress marker) in the hippocampus of aged mice.^51^ Surgical stress resulted in a reduction of SOD activity and an elevation of ROS and MDA levels in the aged mice's hippocampus (Figure 2A–C; *sham* vs. *surgery*, *p <* 0.001 for SOD, *p <* 0.01 for ROS, *p <* 0.001 for MDA, *n =* 5/group), indicating a significant increase in hippocampal oxidative conditions following surgery. Conversely, EA pretreatment boosted SOD activity and diminished ROS and MDA levels in the hippocampus (Figure 2A–C; *sham* vs. *surgery*, *p =* 0.0374 for SOD, *p =* 0.0128 for ROS, *p <* 0.01 for MDA, *n =* 5/group).

High levels of oxidative stress may impair autophagy,^52^ suggested as a potential mechanism behind POCD.^23^ Consequently, we examined the level of p‐mTOR in the hippocampus, considering its inverse correlation with autophagy.^53^ Our data revealed that the p‐mTOR level in the hippocampus increased postoperatively (Figure 2D,E; *sham* vs. *surgery*, *p <* 0.0001, *n =* 5/group). However, this increase was mitigated in EA‐pretreated mice (Figure 2D,E; *surgery* vs. *surgery + EA*, *p <* 0.001, *n =* 5/group). We also assessed the protein levels of autophagy markers, LC3B and Beclin‐1, in the hippocampus.^49^, ^54^ Abdominal surgery decreased the LC3B‐II to LC3B‐I ratio and the Beclin‐1 protein level (Figure 2F–H; *sham* vs. *surgery*, *p <* 0.01 for LC3B‐II to LC3B‐I ratio, *p <* 0.0001 for Beclin‐1, *n =* 5/group), confirming the suppression of hippocampal autophagy in aged mice postsurgery. Conversely, EA‐pretreated mice exhibited a rebound in hippocampal autophagy, reflected by an increased LC3B‐II to LC3B‐I ratio and Beclin‐1 protein level (Figure 2F–H; *surgery* vs. *surgery + EA*, *p =* 0.0111 for LC3B‐II to LC3B‐I ratio, *p <* 0.001 for Beclin‐1, *n =* 5/group).

### 3.3 EA pretreatment alleviates postoperative hippocampal neuroinflammation

**FIGURE 3:**
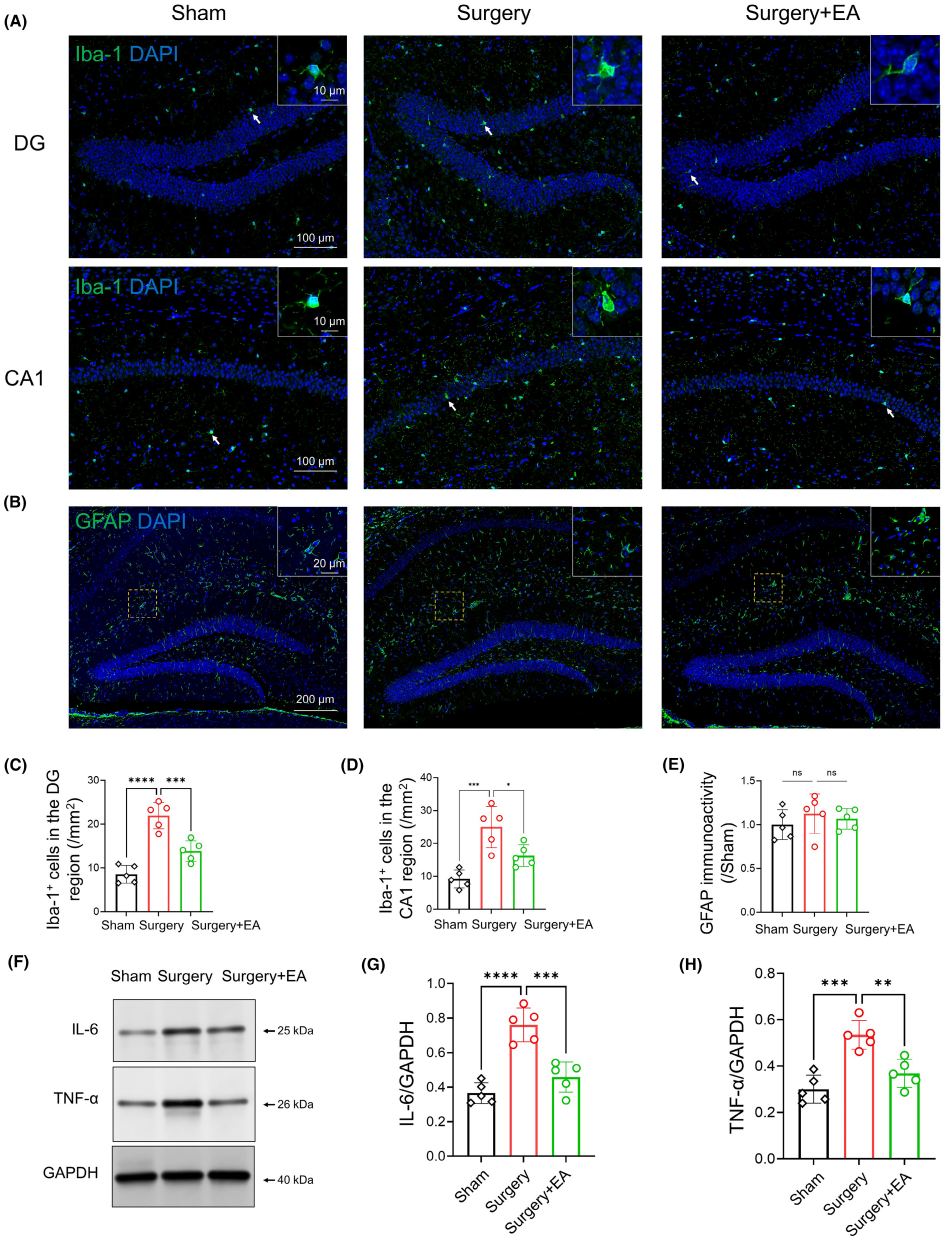
EA Pretreatment Alleviates Postoperative Neuroinflammation in the Hippocampus. (A) Representative images of Iba‐1+ microglia (green) in the hippocampal DG and CA1 regions of aged mice, with nuclei visualized using DAPI (blue). Scale bar = 100 μm. Arrows indicate representative Iba‐1+ microglia (magnified in the top right corner, scale bar = 10 μm). (B) Immunofluorescence images of GFAP (green) in the hippocampus of aged mice on D3, counterstained with DAPI (blue) for nuclei. Scale bar = 200 μm. A highlighted area is framed and magnified in the top right corner (Scale bar = 20 μm). (C, D) The number of Iba‐1+ microglia increased in the hippocampal DG and CA1 regions in the surgery group (*n =* 5/group), but this increase was curtailed in EA‐pretreated mice (*n =* 5/group). (E) Quantitative results for GFAP+ immunoactivity showed no significant difference across the three groups (*n =* 5/group). (F) Protein levels of proinflammatory cytokines (IL‐6 and TNF‐α) were determined by western blot on D3, with IL‐6 and TNF‐α data normalized to GAPDH. (G, H) western blot analysis revealed that surgical stress elevated hippocampal IL‐6 and TNF‐α levels, which were reduced in the EA group (*n =* 5/group). Data are presented as mean ± SD. *p < 0.05, **p < 0.01, ***p < 0.001, ****p < 0.0001. DG, dentate gyrus; GFAP, glial fibrillary acidic protein (astrocyte marker); Iba‐1, ionized calcium‐binding adapter molecule 1 (microglial marker); IL‐6, interleukin 6; TNF‐α, tumor necrosis factor‐α.

To evaluate the effect of EA pretreatment on postoperative hippocampal neuroinflammation, we performed immunostaining of Iba‐1+ (a microglial marker) cells in the hippocampal dentate gyrus (DG) and CA1 regions on D3. The surgery group displayed a higher number of Iba‐1+ microglia in the hippocampal DG and CA1 regions compared to the sham mice (Figure 3A,C,D; *sham* vs. *surgery*, 8.535 ± 2.014 vs. 21.94 ± 3.041 /mm^2^ for DG and 9.212 ± 2.696 vs. 25.0 ± 6.252 for CA1, *p <* 0.0001 for DG and *p <* 0.001 for CA1, *n =* 5/group). However, the EA‐pretreated mice had fewer Iba‐1+ microglia in the DG and CA1 than the surgery group (Figure 3A,C,D; *surgery* vs. *surgery + EA*, 21.94 ± 3.041 vs. 13.83 ± 2.35 /mm^2^ for DG and 25.0 ± 6.252 vs. 16.29 ± 3.295 for CA1, *p <* 0.001 for DG and *p =* 0.0153 for CA1, *n =* 5/group). Given the reported involvement of astrocyte reactivity in postoperative neuroinflammation,^23^, ^55^ we assessed GFAP (an astrocytic marker) immunoactivity in the hippocampus. However, we found no statistically significant difference in GFAP immunoreactivity among the three groups (Figure 3B,E; *F*_(2, 12)_ = 0.6346, *p =* 0.5470, *n =* 5/group).

We also determined hippocampal protein levels of proinflammatory cytokines (IL‐6 and TNF‐α) via western blot. Our data demonstrated that surgical stress caused a marked rise in hippocampal IL‐6 and TNF‐α protein levels (Figure 3F–H; *sham* vs. *surgery*, *p <* 0.0001 for IL‐6 and *p <* 0.001 for TNF‐α, *n =* 5/group). This increase could be mitigated by EA pretreatment (Figure 3F–H; *surgery* vs. *surgery + EA*, *p <* 0.001 for IL‐6 and *p <* 0.01 for TNF‐α, *n =* 5/group).

### 3.4 Intracerebroventricular (i.c.v.) injection of TERT siRNA hinders EA pretreatment‐induced elevation of TERT function

**FIGURE 4:**
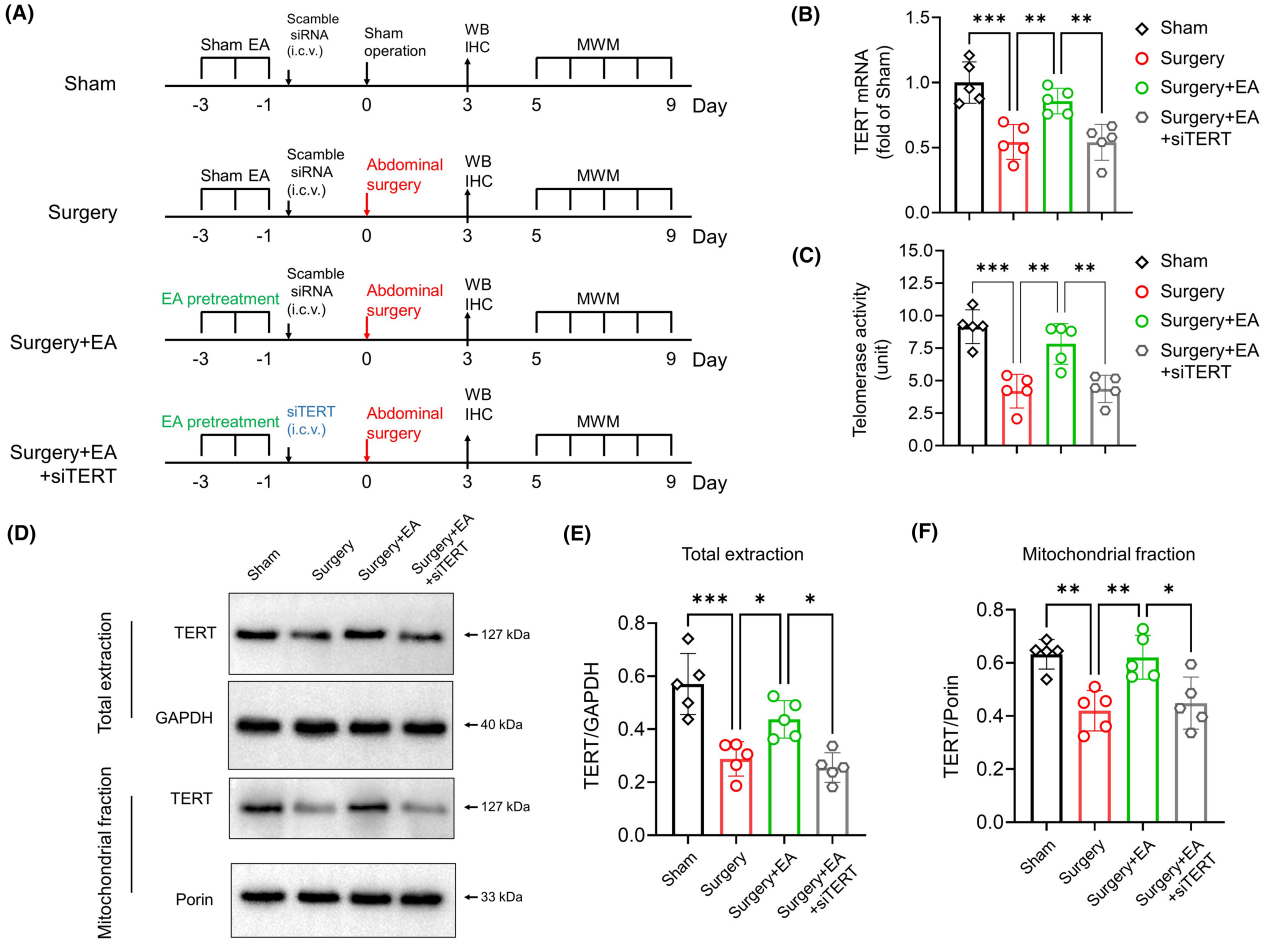
Intracerebroventricular (i.c.v.) Injection of TERT siRNA inhibits the enhancement of TERT function by EA Pretreatment. (A) Schematic of the experimental design for the second session. Mice in the sham and surgery groups received i.c.v. injections of scrambled siRNA (negative control, 400 pmol, 5 μL) 24 h before sham or surgical operation. Mice in the surgery+EA + siTERT group received an i.c.v. injection of TERT siRNA (400 pmol). (B) The mRNA level of TERT was examined by qPCR (*n =* 5/group). (C) Telomerase activity in hippocampal tissues was determined using the TeloTAGGG Telomerase PCR ELISA kit (*n =* 5/group). (D) TERT in total proteins and mitochondrial fractions was evaluated by western blot on D3. Levels of TERT in total proteins and mitochondrial fractions were normalized to GAPDH and Porin, respectively. (E, F) Quantification of hippocampal TERT in total proteins and mitochondrial fractions (*n =* 5/group). Data are presented as mean ± SD. *p < 0.05, **p < 0.01, ***p < 0.001, and ****p < 0.0001. IHC, immunohistochemistry; MWM, morris water maze; TERT, telomerase reverse transcriptase; WB, Western blot.

To probe the role of TERT in the neuroprotective effects of EA pretreatment, aged mice were administered i.c.v. injections of TERT siRNAs (siTERT, 400 pmol) or scrambled siRNAs (negative control) 24 h prior to surgery (Figure 4A). To evaluate the knockdown efficacy of TERT siRNA, we conducted a preliminary study on aged mice (*n =* 4 per group), administering i.c.v. injections of TERT siRNA (200, 400, and 800 pmol) or scrambled siRNA (negative control, 200 pmol). We discovered that administering 200, 400, and 800 pmol TERT siRNA led to a 19.9%, 56.34%, and 59.78% reduction in TERT mRNA, respectively, and a 5.25%, 36.8%, and 38.36% decrease in TERT protein levels in the hippocampus, compared to the negative control (Figure S1). From these preliminary results, we chose 400 pmol as the dose for the following study. This is the lowest effective dose, minimizing potential adverse effects while still achieving the desired knockdown of hippocampal TERT levels.

**FIGURE 5:**
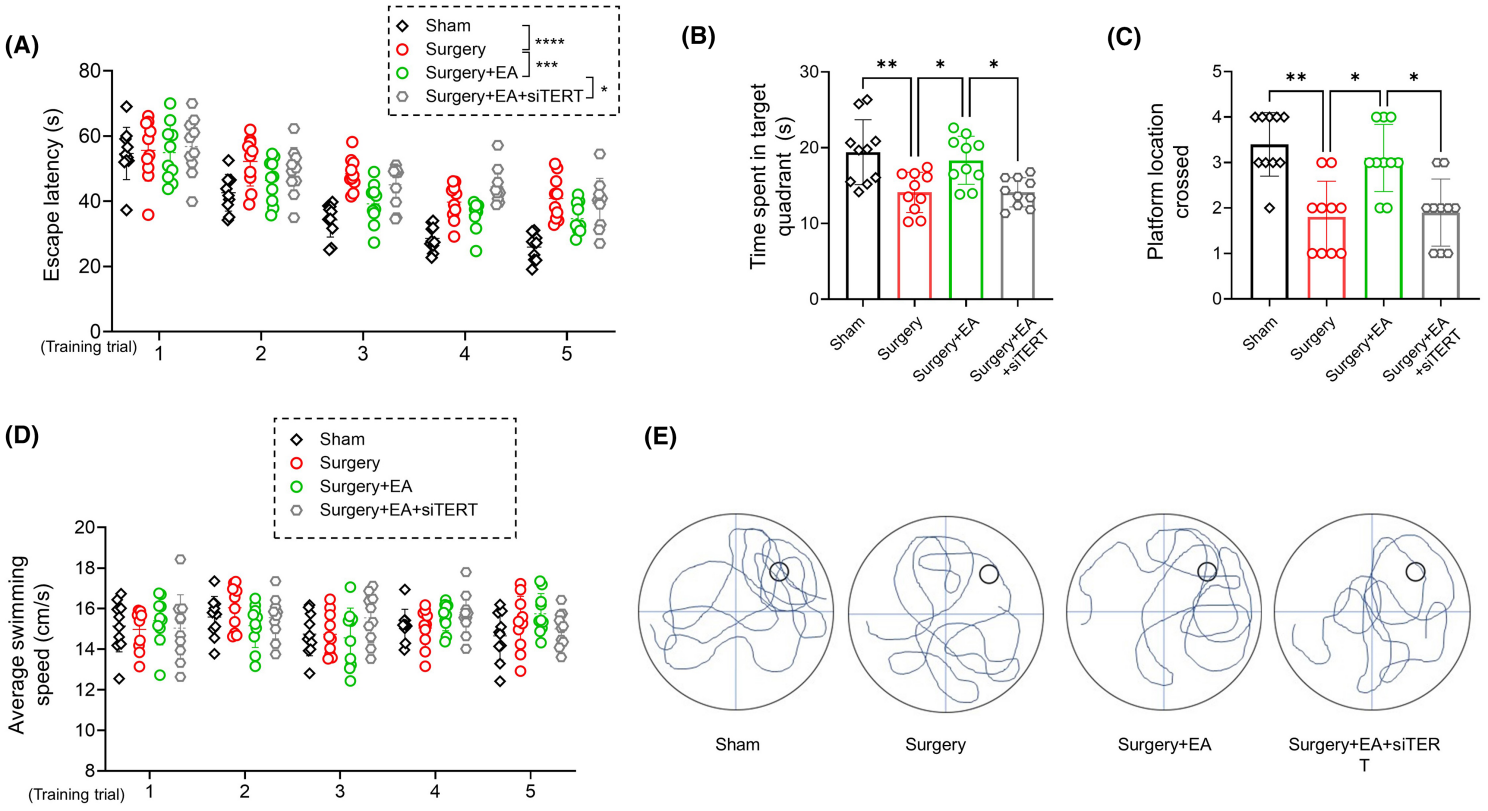
TERT Knockdown Negates the Neuroprotective Effect of EA against Postoperative Cognitive Decline. (A) Escape latency of the 5‐day MWM training trial. Mice in the surgery+EA + siTERT group exhibited a longer latency to reach the hidden platform compared to mice in the surgery+EA group (two‐way ANOVA, *F*_(1, 18)_ = 8.260, *p* = 0.0101 for treatment condition, *n =* 10/group). (B, C) Mice in the surgery+EA + siTERT group spent less time in the target quadrant and crossed the platform location fewer times than those in the surgery+EA group (*p* = 0.0227 for time spent in the target quadrant, *p* = 0.0437 for platform location crossings, *n =* 10/group, Kruskal–Wallis ANOVA test). (D) Average swimming speed of aged mice in MWM training trials (cm/s, *n =* 10/group). (E) Representative trace diagrams in the probe trial. Data are presented as mean ± SD. *p < 0.05 and **p < 0.01. MWM, Morris Water Maze; siTERT, TERT siRNA.

We observed that the increase in TERT mRNA and telomerase activity in EA‐pretreated mice was hindered by siTERT i.c.v. injection (Figure 4B,C; *surgery + EA* vs. *surgery + EA + siTERT*, *p <* 0.01 for TERT mRNA, *p <* 0.01 for telomerase activity, *n =* 5/group). Furthermore, the preservation of total and mitochondrial TERT protein in EA‐pretreated mice was disrupted by TERT knockdown (Figure 4D–F; *surgery + EA* vs. *surgery + EA + siTERT*, *p =* 0.0117 for total TERT, *p =* 0.0162 for mitochondrial TERT, *n =* 5/group). Additionally, TERT knockdown inhibited the improved postoperative cognitive performance in EA‐pretreated mice, as reflected by increased escape latency (Figure 5A; two‐way ANOVA, *F*_(1, 18)_ = 8.260, *p =* 0.0101 for treatment condition, *n =* 10/group), less time spent in the target quadrant (Figure 5B,E; *p =* 0.0227, *n =* 10/group), and fewer platform crossings (Figure 5C,E; *p =* 0.0437, *n =* 10/group, Kruskal–Wallis ANOVA test). During the training trials, there was no statistically significant difference in average swimming speed among the four experimental groups (Figure 5D, two‐way ANOVA, treatment condition, *F*_(3, 36)_ = 0.7810, *p =* 0.5123 for treatment condition, *n =* 10/group).

### 3.5 TERT knockdown exacerbates oxidative damage and disrupts autophagy in the hippocampus of EA‐pretreated mice

**FIGURE 6:**
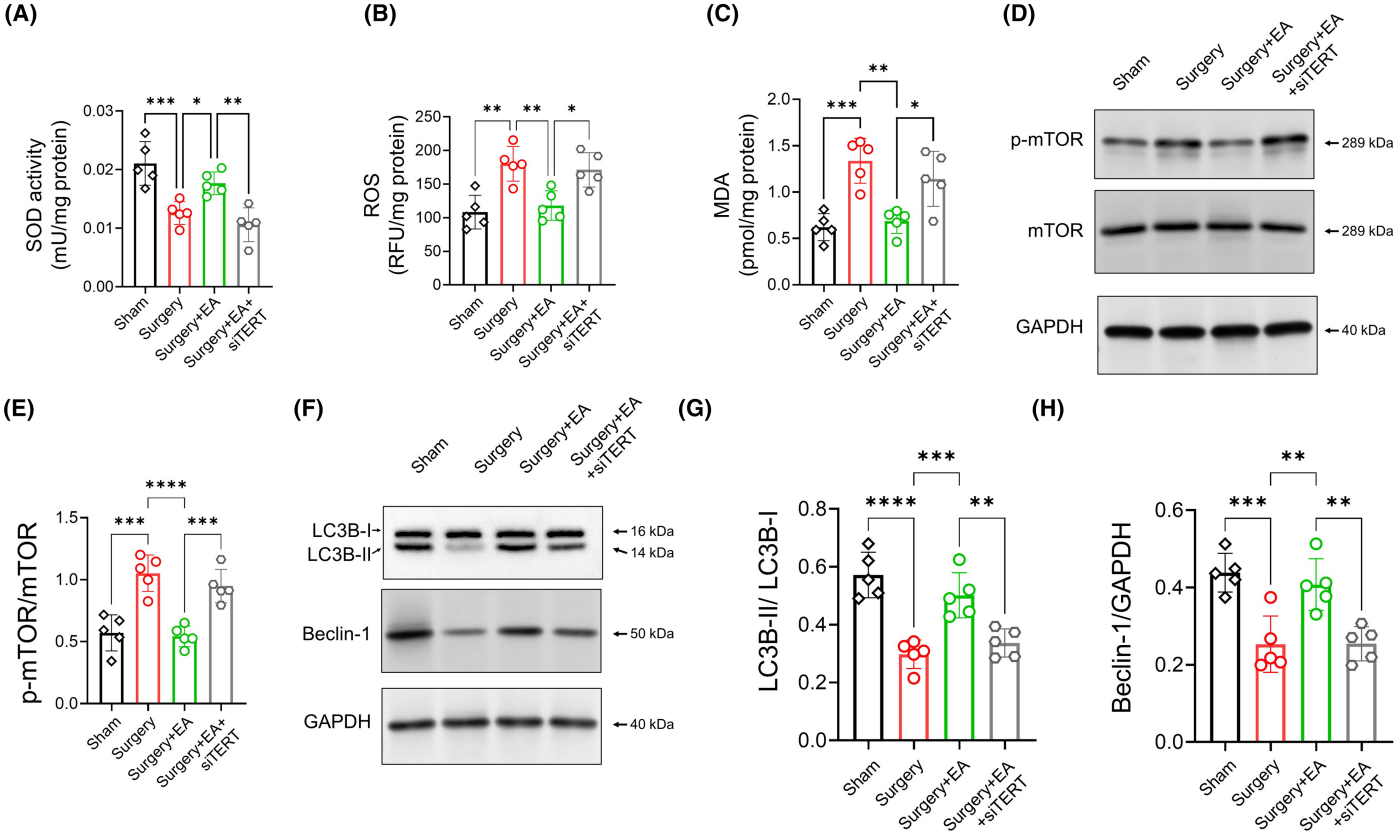
TERT Knockdown Worsens Oxidative Damage and Disrupts Autophagy in the Hippocampus of EA‐Pretreated Mice. (A–C) Oxidative damage in the hippocampus was assessed by testing SOD, ROS, and MDA (*n =* 5/group). (D) p‐mTOR levels, normalized to total mTOR, were measured by western blot on D3. (E) The inhibitory effect of EA pretreatment on p‐mTOR was lessened with TERT knockdown (*surgery + EA* vs. *surgery + EA + siTERT*, *n =* 5/group). (F) Protein levels of autophagy‐related proteins LC3B and Beclin‐1 were analyzed via western blot on D3. The LC3B‐II level was normalized to LC3B‐I, while the Beclin‐1 protein level was normalized to GAPDH. (G, H) The enhanced LC3B‐II/LC3B‐I ratio and Beclin‐1 protein level seen with EA pretreatment was diminished by TERT knockdown (*surgery + EA* vs. *surgery + EA + siTERT*, *n =* 5/group). Data are presented as mean ± SD. *p < 0.05, **p < 0.01, ***p < 0.001, and ****p < 0.0001. LC3B, Microtubule‐associated proteins 1A/1B light chain 3B; MDA, Malondialdehyde; mTOR, Mammalian Target of Rapamycin; SOD, Superoxide Dismutase.

Subsequently, we investigated whether the antioxidative and proautophagic effects of EA pretreatment were due to EA pretreatment's preservation of TERT function. Our findings showed that TERT knockdown via i.c.v. injection of siTERT dulled the restoration of SOD activity and the suppression of ROS and MDA levels in EA‐pretreated mice (Figure 6A–C; *surgery + EA* vs. *surgery + EA + siTERT*, *p <* 0.01 for SOD, *p =* 0.0186 for MDA, *n =* 5/group). Similarly, the proautophagy effect of EA pretreatment was lessened by TERT knockdown, as indicated by a higher p‐mTOR level, reduced LC3B‐II to LC3B‐I ratio, and diminished protein level of Beclin‐1 (Figure 6D–H; *surgery + EA* vs. *surgery + EA + siTERT*, *p <* 0.001 for p‐mTOR, *p <* 0.01 for LC3B‐II to LC3B‐I ratio, *p <* 0.01 for Beclin‐1, *n =* 5/group).

### 3.6 TERT knockdown impedes the anti‐inflammatory effects of EA pretreatment

**FIGURE 7:**
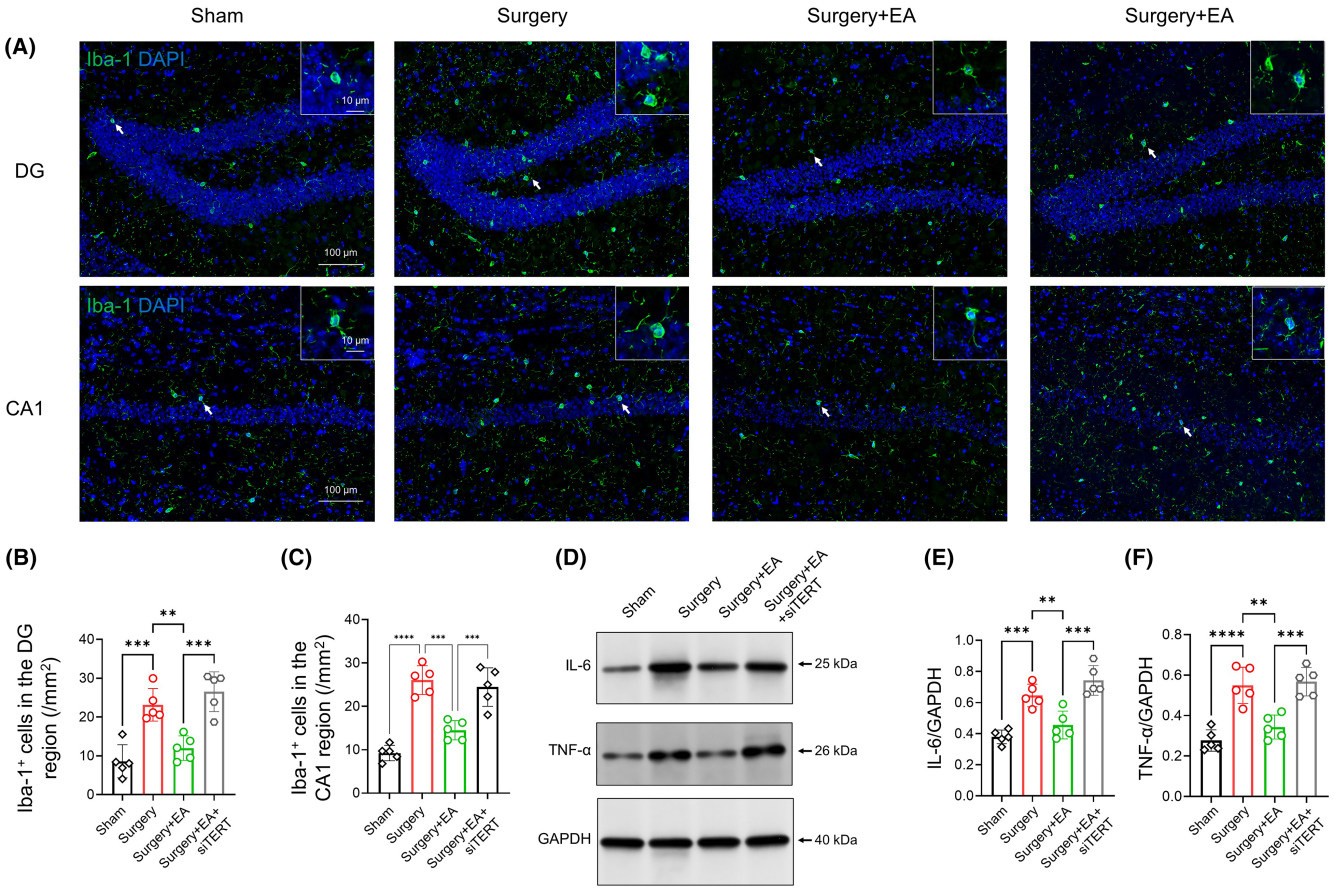
TERT Knockdown Diminishes the Anti‐Inflammatory Impact of EA Pretreatment. (A) Images present Iba‐1+ microglia (green) in the hippocampal DG and CA1 areas of aged mice. DAPI (blue) was used for nucleus visualization. Scale bar = 100 μm. Arrows indicate Iba‐1+ microglia, which are enlarged in the top right corner (scale bar = 10 μm). (B, C) Results showed that TERT knockdown nullified the reduction in Iba‐1+ microglia in the hippocampal DG and CA1 regions brought about by EA (*surgery + EA* vs. *surgery + EA + siTERT*, *n =* 5/group). (D) The protein level of proinflammatory cytokines (IL‐6 and TNF‐α) was measured via western blot on D3. IL‐6 and TNF‐α levels were normalized to GAPDH. (E, F) The EA‐associated reduction in hippocampal IL‐6 and TNF‐α levels was counteracted by TERT knockdown (*surgery + EA* vs. *surgery + EA + siTERT*, *n =* 5/group). Data are shown as mean ± SD. *p < 0.05, **p < 0.01, ***p < 0.001, and ****p < 0.0001. DG, Dentate Gyrus; Iba‐1, Ionized Calcium‐Binding Adapter Molecule 1 (a microglial marker); IL‐6, Interleukin 6; TNF‐α, Tumor Necrosis Factor‐α.

Following this, we aimed to determine whether TERT knockdown would impact the anti‐inflammatory effects of EA stimulation in the POCD model. Immunofluorescence staining of Iba‐1+ in the hippocampal DG and CA1 regions demonstrated that TERT knockdown hindered the reduction of Iba‐1+ microglia in these regions that were induced by EA (Figure 7A–C; *surgery + EA* vs. *surgery + EA + siTERT*, 12 ± 3.154 vs. 26.5 ± 5.121 /mm^2^ and *p <* 0.001 for DG, 14.51 ± 2.23 vs. 24.45 ± 4.44 /mm^2^ and *p <* 0.001 for CA1, *n =* 5/group). Similarly, the inhibitory effect of EA on the production of hippocampal IL‐6 and TNF‐α was also hampered by TERT knockdown (Figure 7D–F; *surgery + EA* vs. *surgery + EA + siTERT*, *p <* 0.001 for IL‐6, *P <* 0.001 for IL‐6, *n =* 5/group).

## 4 DISCUSSION

Augmented oxidative stress is a pivotal factor contributing to brain aging and postoperative cognitive dysfunction (POCD), as underscored by prior research.^10^, ^18^, ^19^ In parallel, the preservation of telomerase activity, particularly telomerase reverse transcriptase (TERT) function, has been suggested as a viable strategy against oxidative cellular damage and the aging process.^25^, ^26^, ^33^ Our research resonates with these assertions, positing that surgical procedures notably suppress hippocampal telomerase activity and TERT function in aged mice, a phenomenon associated with amplified oxidative stress, disrupted autophagy, and heightened neuroinflammation. Conversely, mice receiving electroacupuncture (EA) pretreatment manifested enhanced telomerase activity, elevated TERT protein levels, and increased mitochondrial localization of TERT, hinting at a significant regulatory effect of EA on TERT. Critically, the benefits of EA, which include cognitive improvement and oxidative stress suppression, were nullified upon TERT knockdown. Consequently, our findings emphasize the protective role of TERT function in mitigating hippocampal oxidative stress and neuroinflammation postsurgery in aged mice, potentially mediated via EA pretreatment. This novel understanding of TERT's role in POCD and its contribution to EA's neuroprotective effect could pave the way for new therapeutic approaches.

Aging is an established risk factor for the development of POCD.^4^, ^5^, ^6^ As individuals age, oxidative stress increases,^56^ playing a pivotal role in the emergence of POCD.^24^ TERT, the enzymatic component of telomerase, has been identified as a potential therapeutic target in managing aging‐related disorders.^57^ This knowledge prompted us to investigate the potential role of TERT in surgery‐induced oxidative stress and cognitive dysfunction. Our findings reveal a surgical intervention‐induced decline in both hippocampal telomerase activity and TERT levels. This decline is associated with the onset of oxidative stress, as evidenced by the decreased levels of SOD and increased levels of ROS and MDA in aged mice. We hypothesize that the observed suppression of TERT function postsurgery may be linked to the exacerbated oxidative condition, given that oxidative stress has been shown to directly inhibit TERT function.^58^ In addition to the direct suppression of TERT, our results indicate that surgical interventions decrease the mitochondrial localization of TERT. The translocation of TERT from the nucleus to mitochondria is an established mechanism through which TERT reduces ROS levels^36^, ^58^ and is often considered a defensive response to stress stimuli, such as ischemia/reperfusion injury,^36^ ethidium bromide,^59^ and ultraviolet radiation.^59^ Therefore, the observed reduction of mitochondrial TERT in the hippocampus of aged mice following surgery is somewhat unexpected. It suggests that the regulation of TERT translocation to mitochondria might be complex and context‐dependent, particularly in the aged brain. Miwa et al.^32^ noted a decrease in TERT levels in the brain during aging, accompanied by an increase in ROS released from the mitochondrial electron transport chain. It is conceivable that the age‐related decrease in TERT expression leaves it vulnerable to direct oxidative stress, potentially diminishing its responsiveness to surgical stress in the aged brain.

In addition to the surgery's impact on TERT, our study also highlights a decrease in telomerase activity in the hippocampus of aged mice postsurgery. It is noteworthy that telomerase activity typically diminishes after birth^60^ and is principally preserved within proliferating neural stem cells (NSCs) situated in specific regions of the adult brain,^61^ including the dentate gyrus (DG) of the hippocampus. Prior research indicates that surgical procedures and anesthesia can hinder NSC proliferation and differentiation, as well as compromise the survival of newly formed neurons in the hippocampus.^62^, ^63^ Consequently, the observed decrease in hippocampal telomerase activity in aged mice postsurgery could potentially be attributed to disruptions in NSC activity.

Telomerase activators, such as TA‐65, have been commercially developed to counterbalance aging and oxidative stress by stimulating telomerase activity and promoting an increase in TERT expression.^35^, ^36^, ^64^, ^65^ Meanwhile, EA stimulation, a noninvasive brain modulation technique, has showcased substantial antioxidant impacts across numerous neurological disorders.^45^, ^66^, ^67^ Our study extends these findings and suggests that EA stimulation may offer an alternate route to preserve brain TERT function. We observed that EA‐pretreated mice exhibited reduced hippocampal oxidative stress, increased telomerase activity, and heightened TERT levels postsurgery. Furthermore, EA pretreatment mitigated the surgery‐induced reduction in mitochondrial TERT protein levels, indicating a potential promotive effect of EA on mitochondrial TERT localization. Our findings also revealed that TERT knockdown via siRNA not only reduced TERT levels in the hippocampus of aged mice but also attenuated the antioxidant effects of EA pretreatment. This suggests that TERT might play a key role in the antioxidant properties associated with EA in the context of POCD.

The mechanisms behind the observed upregulation of TERT in the hippocampus of aged mice postsurgery through the application of EA remain largely unexplored. Nevertheless, it is plausible that various key elements, particularly considering the well‐documented influence of neuroinflammatory responses^57^ and oxidative stress^57^, ^68^ on TERT expression and function in the adult brain, could be involved. These factors are recognized as significant contributors to postoperative cognitive decline.^18^, ^69^ Crucially, the neuroprotective effects of EA are largely attributable to its anti‐inflammatory^41^, ^42^, ^43^ and antioxidant^44^, ^45^ effects. It is thought that EA might stimulate the activation of nuclear factor erythroid 2‐related factor 2 (Nrf2),^70^ an antioxidant transcription factor. This could potentially enhance TERT expression through its interaction with the antioxidant response element.^71^ In addition, EA might suppress the activity of NF‐κB,^72^ which is known to inhibit TERT expression by binding to its promoter region.^73^ Therefore, we suggest that EA could improve TERT functionality in the hippocampus of aged mice postsurgery through the selective activation of anti‐inflammatory and antioxidant signaling pathways. Furthermore, by restoring TERT functionality, EA could help mitigate the damaging effects of postoperative hippocampal oxidative stress, as suggested by our research findings. However, these hypothesized mechanisms necessitate further rigorous scientific exploration for confirmation.

Aging‐associated neurodegenerative diseases are characterized by increased oxidative stress, leading to the accumulation of dysfunctional organelles^74^ and proteins,^75^ which further exacerbate neuronal dysfunction or apoptosis. Normally, autophagy processes and degrades these toxic products, protecting neurons from oxidative damage and cell death. However, this protective function of autophagy diminishes with brain aging,^76^, ^77^ rendering neurons more vulnerable to stress stimuli. Our study found that surgical interventions inhibited autophagy in the hippocampus of aged mice, as evidenced by hyperactivated mTOR (a negative regulator of autophagy^53^) and decreased protein levels of autophagy markers (LC3B and Beclin‐1). This finding aligns with Gao et al.'s study,^24^ which demonstrates that upregulation of hippocampal autophagy shields against surgery‐induced synaptic dysfunction and cognitive decline in aged mice. Notably, several studies suggest that EA treatment promotes autophagy induction and ameliorates cognitive dysfunctions in animal models of AD^42^ and PD.^43^ It was thus expected to observe that EA pretreatment counteracted postsurgery suppression of hippocampal autophagy. Increasing evidence has highlighted the role of TERT in mediating autophagy. For instance, TERT overexpression in cancer cells enhances autophagy, while TERT‐deficient cells exhibit impaired autophagic flux.^78^ A recent animal study further shows that increased TERT protein level promotes autophagy and attenuates pathological changes in a mouse PD model.^65^ Building on these findings, our current study revealed that TERT knockdown reduced hippocampal autophagy protein markers and upregulated mTOR in EA‐pretreated mice, suggesting that the preservation of TERT function mediated by EA might underlie its promotional effect on hippocampal autophagy following surgery.

Neuroinflammation mediated by microglia is another key contributor to POCD.^18^, ^19^, ^79^ Within the brain, oxidative stress can induce microglia activation, which in turn exacerbates oxidative damage by releasing ROS and pro‐inflammatory cytokines.^20^ Given the elevated oxidative stress present in the aging brain, this This redlined PDF shows all copy edited changes made to your manuscript. They are for your reference only. Please make all edits in the HTML version of the proofs. phenomenon may partially explain why surgery‐induced neuroinflammation is more pronounced in the aged.^18^, ^19^ Consistent with this, our results revealed that surgical interventions significantly increased microglial proliferation and pro‐inflammatory mediators (IL‐6 and TNF‐α) in the hippocampus. Conversely, EA‐pretreated mice showed a reduction in microglial activation and inflammatory cytokine production post‐surgery. This agrees with the widely acknowledged anti‐inflammatory properties of EA in various neurological conditions, such as AD,^41^ ischemic stroke,^80^ spinal cord injury,^81^ and depression.^82^

Beyond its role in regulating oxidative stress and autophagy, TERT is also involved in modulating inflammatory responses. For example, TERT can function as a target gene of NF‐κB, a well‐established regulator of inflammatory responses, in macrophages exposed to inflammatory stimuli, while TERT deficiency induces a senescent cell phenotype.^83^ Another study revealed that TERT induction in colon organoids from patients with inflammatory bowel disease led to a decrease in DNA damage activation and inflammatory signaling, such as pro‐IL‐18.^84^ Animal studies further corroborated this link, showing that TERT‐deficient mice exhibited reduced neuroinflammation following ischemic brain injury,^85^ and increased TERT protein levels were associated with melatonin‐mediated shifts of inflammatory M1 microglia toward an anti‐inflammatory M2 phenotype.^86^ In line with these findings, our study found that TERT knockdown counteracted the anti‐inflammatory effect of EA stimulation, suggesting a correlation between TERT function and postsurgery neuroinflammation as well as EA‐induced neuroprotection.

While our study illuminates the role of EA in safeguarding hippocampal TERT function in aged mice following surgery, it is essential to acknowledge some limitations to comprehending our results. Our primary constraint lies in detecting changes in TERT expression without determining their specific cellular origin. In the adult brain, TERT expression is across a variety of brain cell types, including neural stem cells^57^ and neurons.^87^ Despite our attempts to utilize immunofluorescence to pinpoint specific hippocampal cells experiencing TERT fluctuations, our antibodies resulted in only nonspecific signals from TERT immunofluorescence staining. Therefore, the TERT modifications detected could potentially be an aggregate effect of alterations in these different cell types. Further research is warranted to definitively determine the cellular origins of these TERT changes. Additionally, the apparent augmentative effect of EA on TERT function and expression may be influenced by TERT's inherent recovery over time. Our qPCR data highlighted a gradual resurgence of TERT expression commencing from the third postoperative day, indicating a potential natural recovery process of TERT functionality. Although we validated superior TERT function in the hippocampus of the Surgery+EA group compared to the Surgery group on the third postoperative day, it does not entirely negate the potential confounding influence of TERT's natural recuperation on the subsequent behavioral outcomes. Therefore, future investigations should consider this potential confounding factor.

In conclusion, our study highlights the pivotal role of maintaining TERT function within the aging brain as a strategy to counteract oxidative stress, impaired autophagy, and neuroinflammation in the context of POCD. Furthermore, our findings uncover a hitherto unrecognized neuroprotective mechanism of EA stimulation, whereby modulation of TERT function helps to alleviate oxidative damage, dysfunctional autophagy, and neuroinflammation. Accordingly, the safeguarding of TERT function via EA intervention could potentially serve as a compelling approach to the management of POCD.

## AUTHOR CONTRIBUTIONS

WW and XC designed the study and provided funding acquisitions, contributing equally to the work. XC, WW, and JGM completed the biochemical experiments. WW and YSL performed the behavioral tests. WQ supervised the study and provided explicit research mentorship; XC and CC prepared the draft manuscript, which was reviewed and edited by WW, YSL, ZG, and RW.

## FUNDING INFORMATION

This study was supported by the National Natural Science Foundation of China (Grant No. 81903976); National Natural Science Foundation of China (Grant No. 82201549); and GuangDong Basic and Applied Basic Research Foundation (Grant No. 2020A1515110863).

## CONFLICT OF INTEREST STATEMENT

The authors declare no conflicts of interest.

## Supporting information


Figure S1.

## Data Availability

The data that support the findings of this study are available from the corresponding author upon reasonable request.
