# Supplementary figures and images for: Electroacupuncture pretreatment preserves telomerase reverse transcriptase function and alleviates postoperative cognitive dysfunction by suppressing oxidative stress and neuroinflammation in aged mice
Source: CNS Neurosci Ther. 2023 Jul 27;30(2):e14373. doi: 10.1111/cns.14373 (PMC10848091; doi:10.1111/cns.14373)

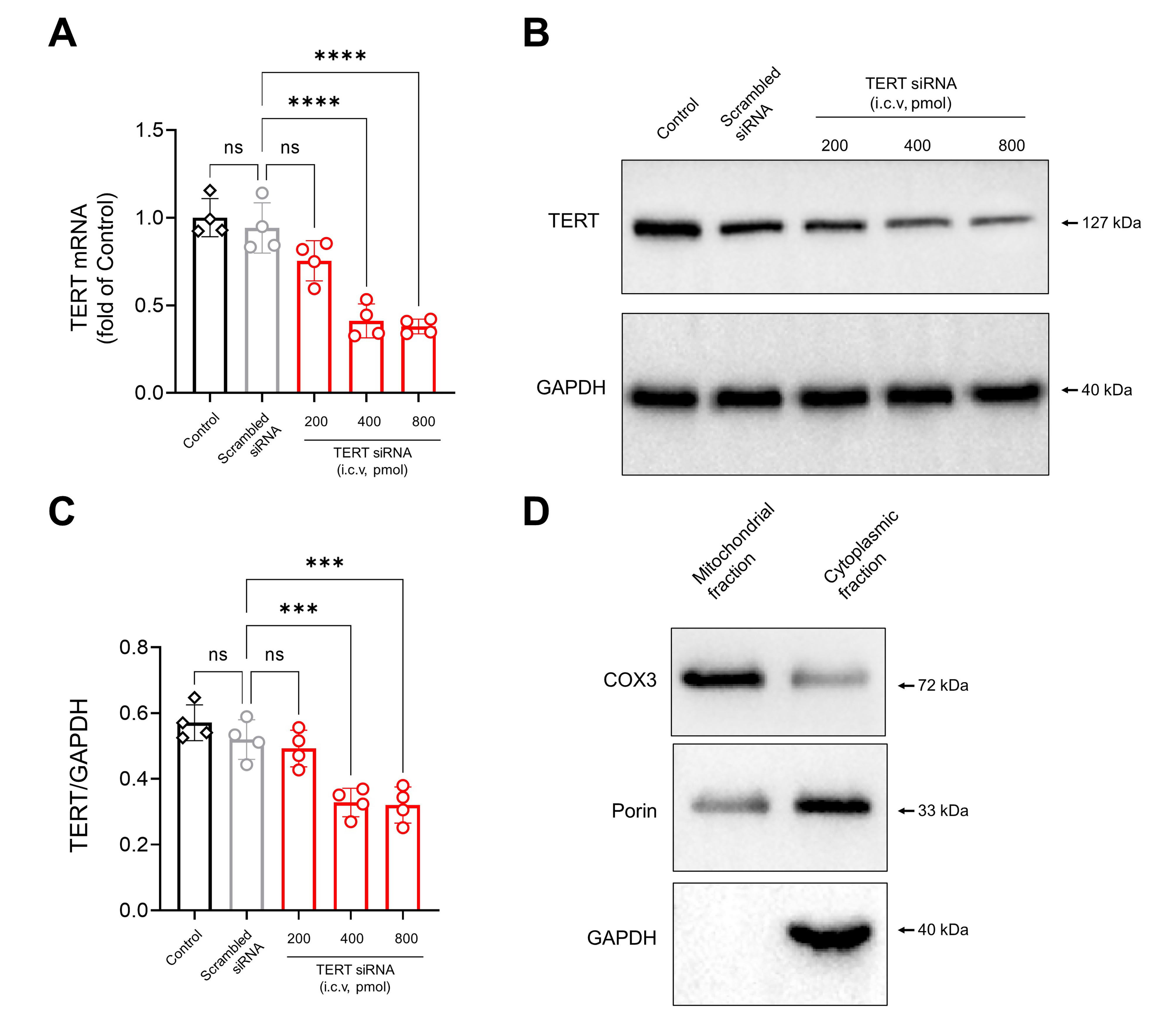

Supplement: Supplementary file 1 — Figure S1. [file CNS-30-e14373-s001.jpg]
